# Retraction: Forecasting the rheological state properties of self-compacting concrete mixes using the response surface methodology technique for sustainable structural concreting

**DOI:** 10.1371/journal.pone.0346403

**Published:** 2026-04-03

**Authors:** The PLOS One Editors

The *PLOS One* Editors retract this article [1,2] due to concerns about potential manipulation of the publication process. These concerns call into question the validity and provenance of the reported results. We regret that the issues were not identified prior to the article’s publication.

KCO did not agree with the retraction. EZ, NV, EMMM, JB, NAEB, NG, and SH either did not respond directly or could not be reached.
